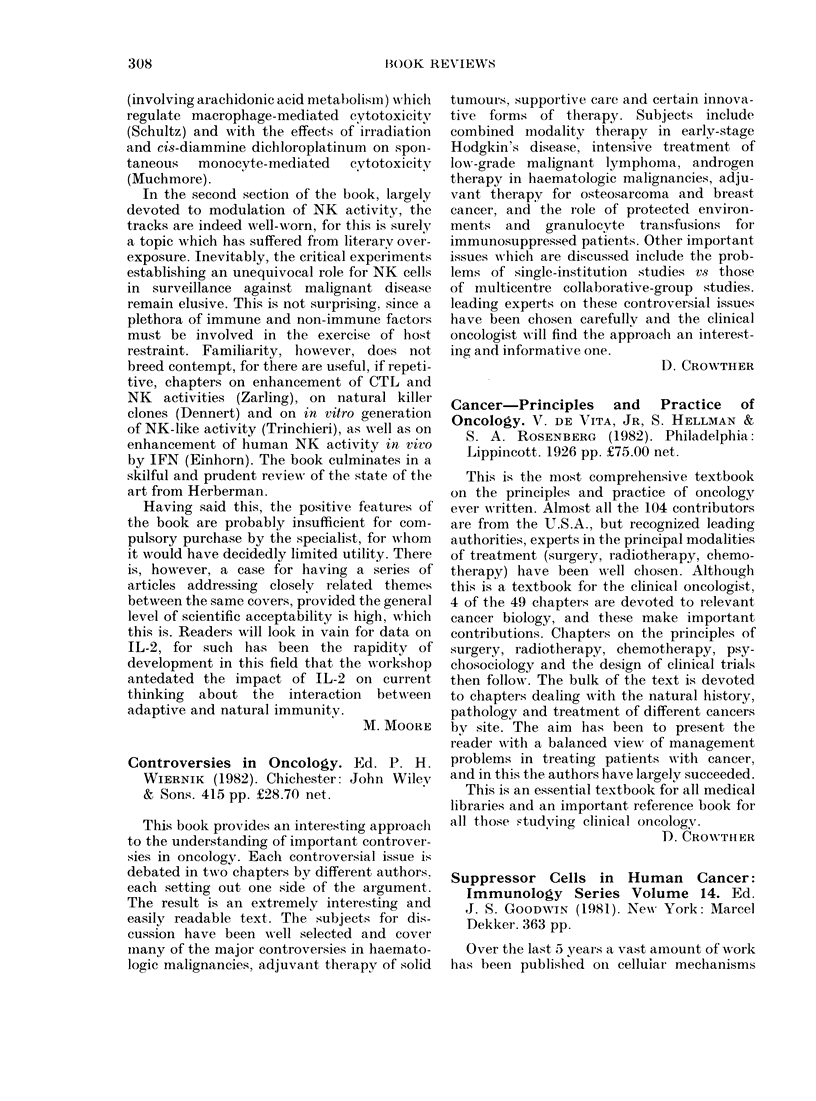# Controversies in Oncology

**Published:** 1982-08

**Authors:** D. Crowther


**Controversies in Oncology.** Ed. P. H. WIERNIK (1982). Chichester: John Wiley & Sons. 415 pp. £28.70 net.

This book provides an interesting approach to the understanding of important controversies in oncology. Each controversial issue is debated in two chapters by different authors, each setting out one side of the argument. The result is an extremely interesting and easily readable text. The subjects for discussion have been well selected and cover many of the major controversies in haematologic malignancies, adjuvant therapy of solid tumours, supportive care and certain innovative forms of therapy. Subjects include combined modality therapy in early-stage Hodgkin's disease, intensive treatment of low-grade malignant lymphoma, androgen therapy in haematologic malignancies, adjuvant therapy for osteosarcoma and breast cancer, and the role of protected environments and granulocyte transfusions for immunosuppressed patients. Other important issues which are discussed include the problems of single-institution studies *vs* those of multicentre collaborative-group studies. leading experts on these controversial issues have been chosen carefully and the clinical oncologist will find the approach an interesting and informative one.

D. CROWTHER